# Design of Economical and Achievable Aluminum Carbon Composite Aerogel for Efficient Thermal Protection of Aerospace

**DOI:** 10.3390/gels8080509

**Published:** 2022-08-17

**Authors:** Yumei Lv, Fei He, Wei Dai, Yulong Ma, Taolue Liu, Yifei Liu, Jianhua Wang

**Affiliations:** 1 CAS Key Laboratory of Mechanical Behavior and Design of Materials, Department of Thermal Science and Energy Engineering, University of Science and Technology of China, Hefei 230027, China; 2 Beijing Institute of Astronautical Systems Engineering, No.1 South Dahongmen Road, Fengtai District, Beijing 100071, China

**Keywords:** aluminum carbon composite aerogel, simple fabrication method, thermal stability, thermal conductivity, heat insulation, numerical simulation

## Abstract

Insulation materials play an extremely important role in the thermal protection of aerospace vehicles. Here, aluminum carbon aerogels (AlCAs) are designed for the thermal protection of aerospace. Taking AlCA with a carbonization temperature of 800 °C (AlCA–800) as an example, scanning electron microscopy (SEM) images show an integrated three-dimensional porous frame structure in AlCA–800. In addition, the thermogravimetric test (TGA) reveals that the weight loss of AlCA–800 is only ca. 10%, confirming its desirable thermal stability. Moreover, the thermal conductivity of AlCA–800 ranges from 0.018 W m^−1^ K^−1^ to 0.041 W m^−1^ K^−1^, revealing an enormous potential for heat insulation applications. In addition, ANSYS numerical simulations are carried out on a composite structure to forecast the thermal protection ability of AlCA–800 acting as a thermal protection layer. The results uncover that the thermal protective performance of the AlCA–800 layer is outstanding, causing a 1185 K temperature drop of the structure surface that is exposed to a heat environment for ten minutes. Briefly, this work unveils a rational fabrication of the aluminum carbon composite aerogel and paves a new way for the efficient thermal protection materials of aerospace via the simple and economical design of the aluminum carbon aerogels under the guidance of ANSYS numerical simulation.

## 1. Introduction

With the increasing speed and flight time of aerospace vehicles, the high heat fluxes arising from aerodynamics and combustion have created a growing demand for insulation materials to protect the key components of aircrafts [1,2]. However, the heat resistance, thermal stability, heat insulation ability and weight of current insulation materials are still unable to satisfy the growing needs of aerospace thermal protection [3]. Therefore, it is necessary to explore novel materials with extremely light weight and outstanding thermal insulation performance at ultrahigh temperatures [4].

A tremendous amount of research is currently focused on carbon-based materials, which have the ability to withstand ultrahigh temperatures up to 3000 °C [5], making them the most promising candidates for lightweight aerospace materials [6]. Carbon aerogel (CA) possesses an abundant mesoporous and superimposed nanoparticle network, which confers it various unique properties, such as high specific surface area and extremely low density [7]. Besides, the carbon skeleton of the carbon aerogel has ultralow thermal conductivity [8], and the heat transfer through the gases is suppressed due to the nanoscale pores in the carbon aerogel [9]. Compared with other aerogels, the higher specific extinction coefficient of the carbon aerogel could significantly reduce the radiation heat transfer between it and the high temperature environment [10]. Overall, the carbon aerogels provide enormous potential for acting as a barrier in convection, conduction and radiation heat transfer. Furthermore, carbon aerogels are able to keep their mesoporous structure in an inert atmosphere above 2000 °C [11], so it appears to be one of the high temperature insulating materials with excellent thermal stability. Hence, developing the carbon aerogel is an effective way to address the problem of thermal protection under high temperatures [12].

Traditional carbon aerogels are fabricated by chemical organic precursors, such as hydroxybenzene, aldehyde, polyimide and polyimide, etc., which does not conform to the concept of green production [13]. Besides, the surface tension of the carbon aerogels is large during the preparation; thus, the pores easily collapse in the drying process [14]. There will also be the obvious expansion or contraction of the carbon aerogel in the carbonization process, which may lead to cracks in the aerogel [15]. Therefore, traditional carbon aerogel fabrication usually requires a complicated solution exchange process and high-cost supercritical drying process to reduce the surface tension and prevent the pore collapse [16], severely limiting the large-scale production and practical applications. To overcome this issue, researchers try to strengthen the gel skeleton with carbon fibers, carbon nanotubes and ceramic fibers as a remedy of those hindrances [17]. Unfortunately, the strong shrinkage mismatch between different structures will generate internal tensile stress [11,18], which still inevitably results in microcracks. Meanwhile, the addition of some fibers may lead to an increase in the thermal conductivity of the carbon aerogel [19], causing a contradiction between the heat insulation ability and mechanical properties. Hence, under the purpose of widely employing the carbon aerogel as thermal protection materials for aerospace applications, the raw materials with low cost, large-scale production and simple fabrication methods should be adopted in the design of the carbon aerogel [20,21]. Moreover, some materials ought to be added to address the problems of cracks and collapse in the production process of carbon aerogel, and at the same time the addition of these materials should not bring about other problems [22].

Starch is an abundant resource in the leaves and seeds, which could be widely produced from the nature [23]. In addition, the starch easily forms colloidal solutions at high temperatures under the high temperature [24]. Subsequently, hydrogels can be converted from the colloidal solution by reforming the hydrogen bonds during the decrease in temperature [25]. Hence, the starch exhibits prominent advantages as the raw material to fabricate the carbon aerogels, since it does not require a complex pre-treatment or additional post-processing step [26,27]. Moreover, the introduction of aluminum ions in the process of the fabrication of carbon aerogel has been proposed as the way to promote the polymerization of organic precursors [28]. Meanwhile, aluminum oxide has a certain effect on restraining the expansion or contraction of the carbon aerogel in the carbonization process [29]. Hence, the aluminum could promote the development of a three-dimensional porous frame structure in the carbon aerogel.

Herein, carbon aerogels are designed and fabricated using starch as a raw material for availability and economy. Meanwhile, aluminum ions are anchored on the carbon skeleton to improve the cracking during the process of drying or the carbonization process and ensure the integrity of the pores of the carbon aerogel. Taking an aluminum carbon composite aerogel (AlCA) with a carbonization temperature of 800 °C (AlCA–800) as an example, the scanning electron microscopy (SEM) image displays an unbroken three-dimensional porous frame structure in AlCA–800, which demonstrates that the aerogel is successfully prepared. In addition, elemental mapping images (EDS) exhibit a homogeneous distribution of C and Al_2_O_3_ in AlCA–800. Furthermore, the thermogravimetric test (TGA) indicates that the weight loss of AlCA–800 is only ca. 10%, further confirming its thermal stability. Moreover, the thermal conductivity of AlCA–800 ranges from 0.018 W m^−1^ K^−1^ to 0.041 W m^−1^ K^−1^, revealing a superior heat insulation ability. In addition, ANSYS numerical simulations are carried out on a plate protected with a thermal protection layer made of AlCA–800. The results demonstrate that the thermal protection performance of AlCA–800 layer is desirable, causing a 1185 K temperature drop to the plate surface that was exposed to heat environment for ten minutes. Overall, this work unveils a rational fabrication of the aluminum carbon composite aerogel and paves a way for thermal protection materials with light weight and low thermal conductivity for aerospace applications. Meanwhile, the ANSYS numerical simulation is brought into the design of the materials for predicting the effect of thermal protection in practical applications, which makes the work more reliable and economical.

## 2. Results and Discussion

### 2.1. The Structure and Thermochemical Property of Carbon Aerogels

**Figure 1:**
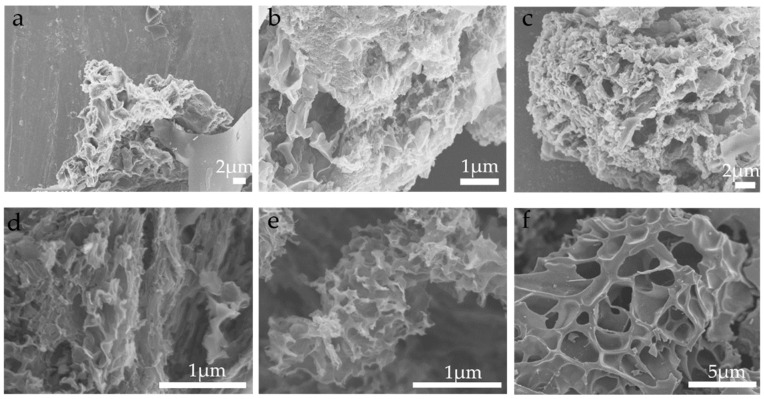
SEM images for (**a**) CA–600, (**b**) CA–800, (**c**) CA–1000, (**d**) AlCA–600, (**e**) AlCA–800 and (**f**) AlCA–1000.

In this work, carbon aerogels (CA) and aluminum carbon composite aerogels (AlCA) were designed and fabricated as effective thermal protection material for aerospace applications, and by altering the carbonization temperature and time, the most suitable aerogel was finally selected. Scanning electron microscopy (SEM) images in Figure 1 revealed the three-dimensional porous frame structure of the CAs and AlCAs. One can observe that the pore structures of CAs and AlCAs can be readily adjusted by increasing the carbonization temperature and time. In addition, the integrity of micropores in CAs was not a patch on AlCAs due to the structural shrinkage and cracking during the carbonization process [30]. Among AICAs, the AlCA–800 has the most excellent three-dimensional porous frame structure, while some holes were sightless and not fully formed in the AlCA–600 due to insufficient carbonization temperature and time [31]. Despite the slight cracks and fractures, AlCA–1000 still retained the three-dimensional porous frame, which indicated the thermal stability of the carbon skeleton. The BET surface, adsorption average pore diameter and quantity adsorbed were displayed in Table 1, further confirming the three-dimensional porous framework in the CAs and AlCAs [32]. Meanwhile, the quantity adsorbed of AlCA–800 was largest, corresponding to the result of SEM images.

**Figure 2:**
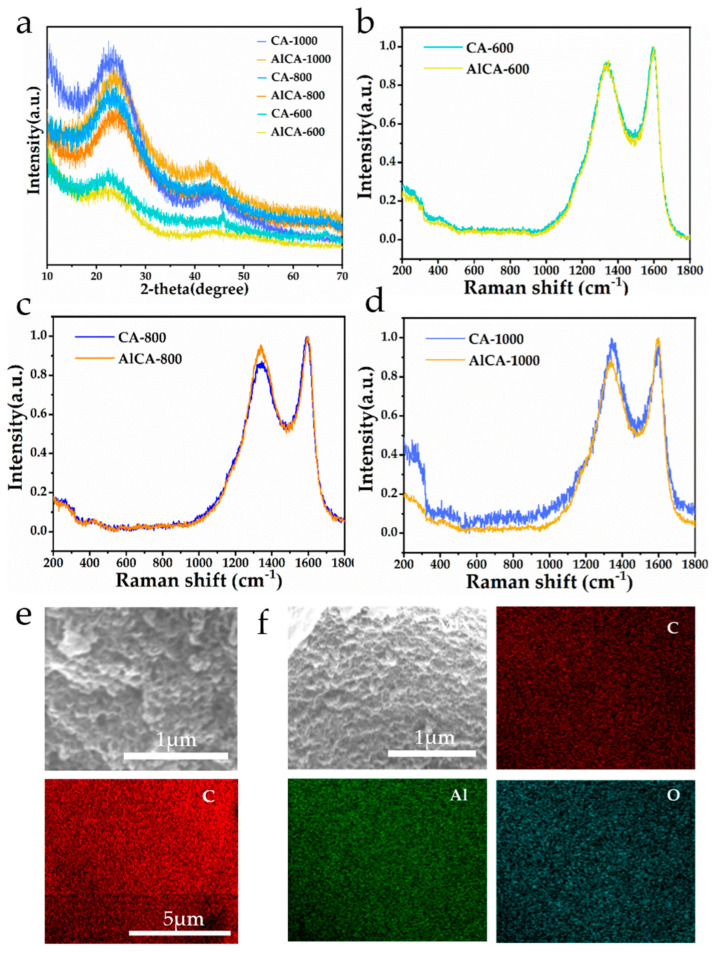
(**a**) XRD pattern for CAs and AlCAs, Raman spectra for (**b**) CA–600 and AlCA–600, (**c**) CA–800 and AlCA–800, (**d**) CA–1000 and AlCA–1000, EDS mapping for (**e**) CA–800 and (**f**) AlCA–800.

To study the elemental composition of the samples, the X-ray diffraction (XRD) pattern was employed for the characterization of the CAs and AlCAs. As shown in Figure 2a, it can be observed that the XRD peak positions of the CAs and AlCAs were consistent but the peak intensities differed greatly, indicating that the substrate of aerogels was graphite [33] (JCPDS card No. 87-0722). However, the XRD peak of the aluminum was not detected due to the small quantity of aluminum. In addition, the Raman spectra of CA–600 and AlCA–600 in Figure 2b demonstrated that there were two characteristic peaks at wavelength 1347 cm^−1^ and 1585 cm^−1^, which could be assigned to graphite D peak and G peak, respectively [34]. The peaks of CA–600 did not demonstrate a detectable difference from those of AlCA–600 due to the weak interaction between the lower temperature of Al and C, corresponding to the result of the XRD patterns (Figure 2a). In addition, Figure 2c revealed the discrepancy of the D peak in CA–800 and AlCA–800, which could be ascribed to the interaction of the Al and C and the decrease in the graphitization degree in AlCA–800 [34,35]. Moreover, as discovered in Figure 2d, the difference appeared in the CA–1000 and AlCA–1000 due to the same reason. In addition, the elemental mapping images (EDS) in Figure 2e further revealed the carbon skeleton in CA–800. A homogeneous distribution of C and Al_2_O_3_ in AlCA–800 was depicted in Figure 2f, confirming that the aluminum was successfully anchored on carbon. As mentioned above, all the results suggested that the CAs and AlCAs were successfully synthesized through the simple sol-gel method.

**Figure 3:**
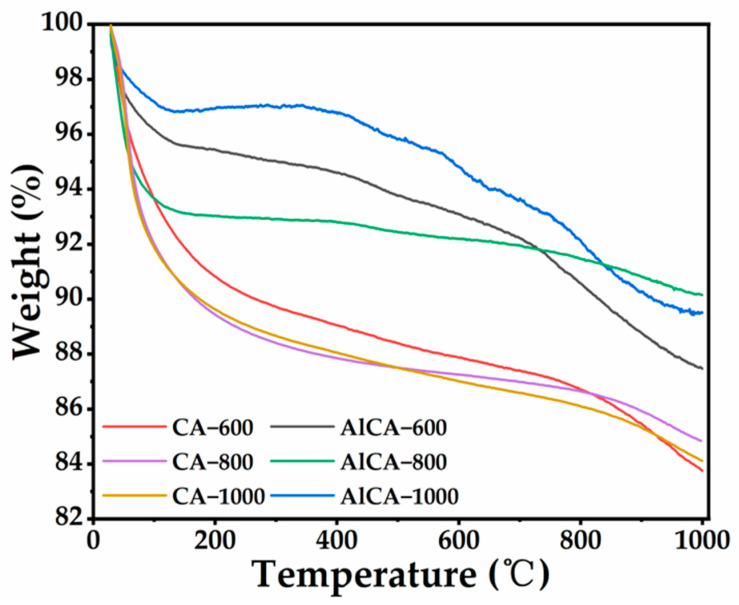
TGA curves of aerogels.

Thermogravimetric tests (TGA) were carried out to reveal the thermal stability of CAs and AlCAs. As shown in Figure 3, in the heating process from 30 °C to 300 °C, the removal of absorbed H_2_O trapped in the samples led to a significant weight loss [36]. Then, there was a slight weight loss of the samples from 300 °C to 600 °C, which was mainly because of the oxidation of a small amount of organic residues [37]. After this stage, a significant weight loss of the carbon CAs and AlCAs occurred in the heating process from 600 °C to 1000 °C, which was attributed to the loss of carbonaceous residuals during depolymerization and decomposition [38]. Based on the TGA curves, the thermal stability of AlCAs was superior to the CAs, which indicated that the Al_2_O_3_ could promote the thermal stability of the aerogel [39]. In addition, the weight loss of AlCA–800 was only ca. 10%, and its thermal stability was better than that of other AlCAs. Moreover, the AlCA–1000 exhibited a favorable thermal stability, which meant that the AlCAs could be employed under higher temperatures. Nevertheless, due to the deficiency of carbonization temperature and time, there was not a complete three-dimensional porous frame structure in AlCA–600, and thus the thermal stability of AlCA–600 was poorer. Overall, the AlCAs had higher thermal stability; in particular, the AlCA–800 possessed excellent thermal stability, demonstrating a great potential to serve as a kind of efficient thermal protective material.

**Figure 4:**
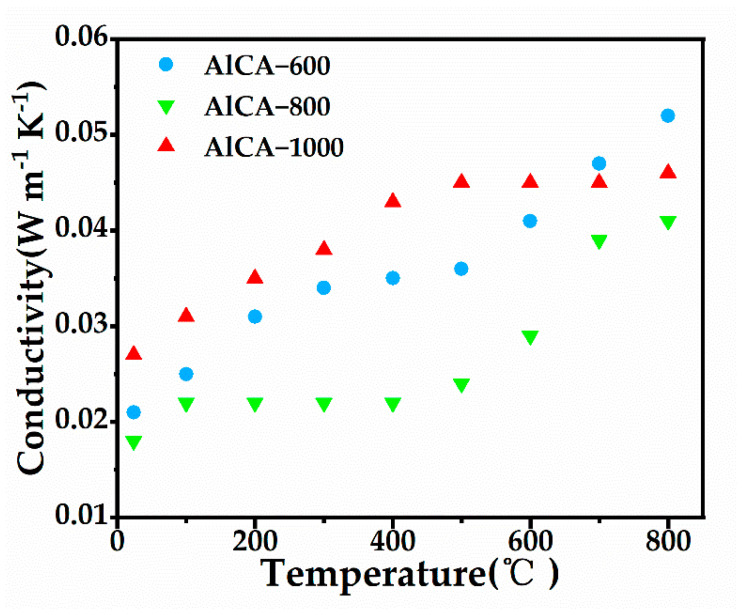
Thermal conductivity of AlCAs.

To investigate the thermal protection ability of the AlCAs, the thermal conductivities at different temperatures were tested. The density of AlCAs was displayed in Table 2. As shown in Figure 4, the thermal conductivity of AlCA–800 was the lowest, ranging from 0.018 W m^−1^ K^−1^ to 0.041 W m^−1^ K^−1^, which could be attributed to the integrated three-dimensional porous frame structure. In addition, the AlCA–1000 demonstrated higher thermal conductivity at low temperatures, but the thermal conductivity presented a slow upward trend as the temperature increases, revealing a fine heat insulation ability under higher temperature. Due to the incomplete three-dimensional network structure, the AICA–600 always demonstrated a relatively higher thermal conductivity. To sum up, the AlCA–800 possessed desirable thermal stability and heat insulation capacity simultaneously, and hence AlCA–800 could be adopted in the thermal protection of aerospace.

### 2.2. The Thermal Protection Performance of Carbon Aerogel

**Figure 5:**
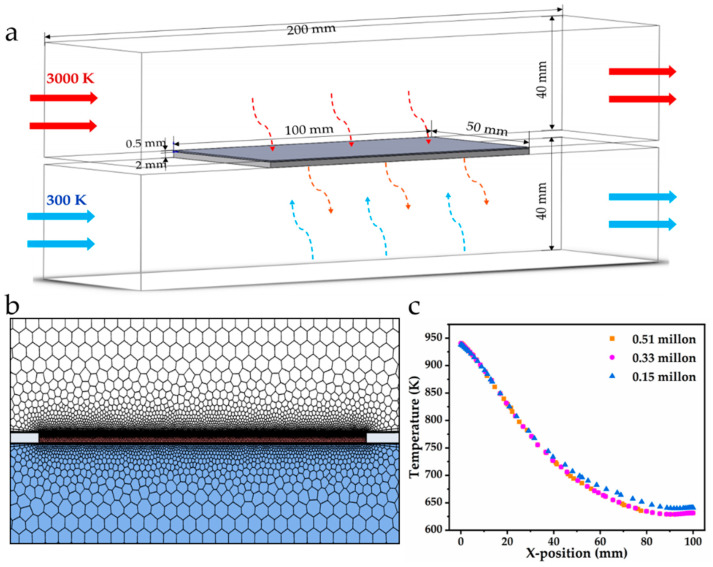
(**a**) Calculation domain and boundary conditions of structure; (**b**) unstructured meshes; (**c**) mesh independence verification.

To explore the thermal protection performance of AlCA–800 as the thermal protection layer, the numerical simulations were performed on a plate with the thermal protection layer (combined structure) under the practical working condition of the scramjet engine (Figure 5a). Meanwhile, the plate without a thermal protection layer (single structure) was also numerical simulated for comparison. The combined structure of the plate to be protected and the thermal protection layer made of AlCA–800 were illustrated in Figure 5a. The plate was initially placed at the ambient environment. Then, its upper surface was exposed to a mainstream with 3000 K high temperature, and its lower surface was cooled by the air with 300 K. The thickness of the plate was 2 mm, while the thickness of carbon aerogel thermal protection layer was 0.5 mm. The TC4 titanium alloy with the thermal conductivity of 7.4 W m^−1^ K^−1^ was adopted as the material of the plate. For the thermal protection layer, the material parameters were customized using the experimental values of AICA–800. In order to reduce the computational region and thereby save computing resource, the periodic boundary conditions were employed on both sides. The numerical simulation was carried out through ANSYS Fluent, and the plate and the hot mainstream were coupled and solved with a transient state pressure-based solver. A second order upwind scheme was employed for the discretization. When the residual of continuity, mass, energy and momentum equations were all lower than 10^−6^, the numerical calculations were considered convergent.

Table 3 displayed the mathematical models describing the heat transfer between the high temperature mainstream and the structure. The fluid flow was calculated by the Menter’s two-equation SST k-ω model [40], of which the veracity was confirmed by many practical applications. Meanwhile, the heat conduction appeared in the thermal protection layer and the lower plate, and thus the Fourier’s law was used to describe the energy balance. Mainstream and cooling air were regarded as ideal gases, and the corresponding viscosity was determined by Sutherland’s formula. Besides, the NIST could serve as the reasonable method to calculate other gas property parameters and fit them with a polynomial formula.

Figure 5b showed the unstructured grid generated in the local calculation region. The grid near the wall was refined due to strong energy exchanges in the fluid near the wall. Besides, the height of the first layer of the grids was set as 1 × 10^−5^ m to ensure the dimensionless wall distance y+ was less than 1, and the grid growth rate was set to 1.2 in this paper. The grid establishment method and distribution directly affected the accuracy of the results, and hence the grid independence of the numerical results was tested through three grid strategies, respectively. The temperature on the centerline of the plate after heating for 100 s was selected for monitoring and comparison. As shown in Figure 5c, the results obtained by the grid sizes of 0.33 million and 0.51 million were similar. Considering the accuracy of the calculation results and the calculation load, a grid size with 0.33 million was adopted in the following calculations.

**Figure 6:**
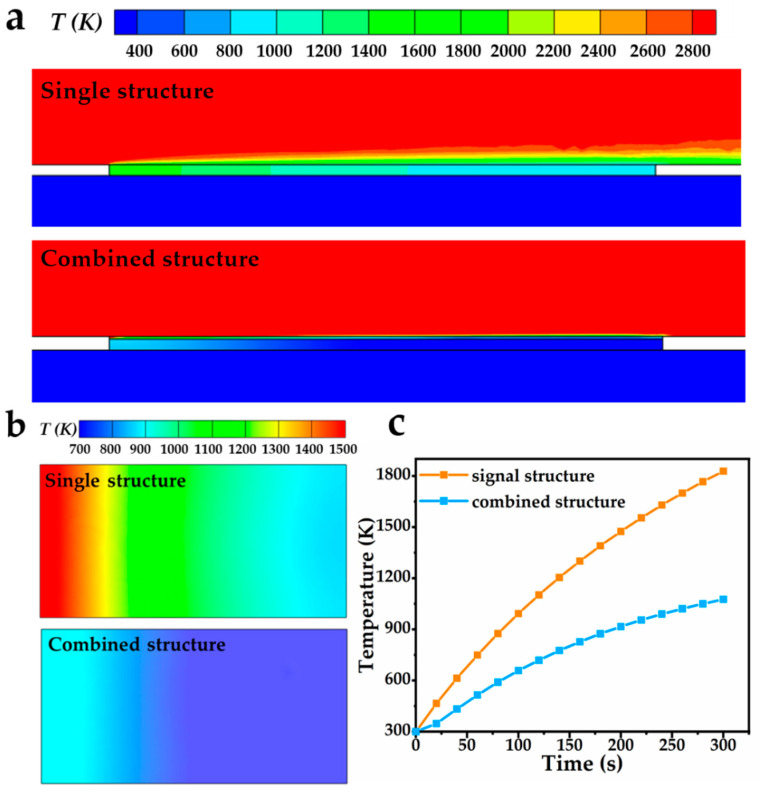
(**a**) Temperature distribution on the lengthwise section at the centerline of two structures; (**b**) temperature distribution on the surface of the plates; (**c**) temperature variation of the plate surfaces.

The temperature distributions on the lengthwise section at the centerline of the two structures were shown in Figure 6a, the heat transfer from the high temperature mainstream to the plate was greatly hindered in the combined structure due to the extremely low thermal conductivity of the AlCA–800. Thus, there was a large temperature drop between the thermal protection layer and the plate. Figure 6b displayed the contours of temperature on the surface of the plates in two structures after 60 s of heating; it could be obviously observed that the surface temperature of the plate in the combined structure was lower, and the temperature distributed more uniformly. Besides, Figure 6c showed the variation of the temperature on the plate surface with heating time, one could see that the rate of the temperature rise slowed down for both structures, while the temperature differences between the two structures were becoming more and more significant. Notably, the temperature on the plate surface of the combined structure was only 1268 K after heating for 10 min under the protection of thermal protection layer, whereas that of the single structure bore the high temperature up to 2453 K, indicating the excellent heat insulation ability of AlCA–800 thermal protection layer. Therefore, the AlCA–800 could be employed as a competitive material for the thermal protection layer, and provided efficient thermal protection for the aerospace vehicles.

## 3. Conclusions

In summary, the aluminum carbon composite aerogels (AlCAs) were designed for realizing the effective thermal protection of aerospace vehicles. The AlCAs were fabricated with the starch, which had a large quantity and low price, being raw, and the preparation process was simple and achievable, guaranteeing the economy of the materials. In addition, the carbon aerogels (CAs) were also fabricated for comparison. The SEM images demonstrated a tightly three-dimensional porous frame structure in the AlCAs, which indicated the successful synthesis of the aerogel. Notably, the AlCA–800 possessed the most desirable three-dimensional porous frame structure due to appropriate carbonization temperature and time. Elemental mapping (EDS) images unveiled a homogeneous distribution of C and Al_2_O_3_ in AlCAs. Moreover, the TGA revealed the lower weight loss of AlCAs than CAs, which could be attribute to the addition of Al. In particular, the weight loss of the AlCA–800 was only ca. 10%, powerfully confirming its thermal stability. Importantly, the thermal conductivity of the AlCA–800 ranged from 0.018 W m^−1^ K^−1^ to 0.041 W m^−1^ K^−1^, which was far below the existing thermal protection materials, meaning that the AlCA–800 had an outstanding heat insulation ability under high temperature. Furthermore, the numerical simulation was carried out on the plate with thermal protection layer made of AlCA–800, aiming at evaluating the thermal protection performance of the AlCA–800. The results uncovered that the thermal protective performance of the AICA–800 layer was extraordinary, causing a 1185 K temperature drop to the plate surface, which was exposed to a heat environment for ten minutes. Consequently, this work not only paved a way for the simple and low-cost fabrication of thermal protection materials with light weight and low thermal conductivity, but also brought ANSYS numerical simulation for predicting its protection performance in practical application situation, which made the work more reliable and economical.

## 4. Materials and Methods

### 4.1. Preparation of Aluminum Carbon Composite Aerogel

Aluminium chloride (AlCl_3_) and soluble starch were obtained from Sinopharm Chem. Reagent Co. Ltd. (Shanghai, China).

Taking carbon aerogel with a heating temperature of 800 °C as an example, the preparation process was as follows: Initially, 50 mL ultrapure water was heated to 80 °C and the 15 g/20 mL starch aqueous solution was added to the water under vigorous stirring for 5 min, and the hydrogels were obtained. Afterwards, the aerogels were obtained after freeze drying. Finally, the aerogels were carbonized at 800 °C for 4 h with a ramping rate of 2 °C min^−1^ under an Ar atmosphere.

Taking aluminum carbon composite aerogel with heating temperature 800 °C as an example, the preparation process was as follows: Initially, 10 mmol AlCl_3_ was dissolved in the 50 mL ultrapure water, the suspension was heated to 80 ℃ under vigorous stirring and the hydrosols were obtained. Then, the 15 g/20 mL starch aqueous solution was added to the above suspension under vigorous stirring for 5 min, and the hydrogels were obtained. Afterwards, the aerogels were obtained after freeze drying. Finally, the aerogels were carbonized at 800 °C for 4 h with a ramping rate of 2 °C min^−1^ under the Ar atmosphere.

### 4.2. Characterization of Aluminum Carbon Composite Aerogel

SEM images were measured on a FEI Sirion-200 (FEI NanoPorts, Hillsboro, ‎OR, USA). XRD patterns were performed on a Rigaku D/MAX-TTRIII diffractometer (Rigaku Corporatio, Tokyo, Japan) with Cu Kα radiation (λ = 1.54178 Å). Raman spectra was acquired by a JY LabRamHR Evolution (HORIBA Jobin Yvon, Palaiseau, France) with a 532 nm laser. SDT Q600 (TA Instruments, New Castle, DE, USA) thermal analyzer was employed to acquire TGA curves under nitrogen atmosphere. Thermal conductivity was determined by the NETZSCH LFA457 thermal analyzer (NETZSCH, Selb, Germany). BET surface area was acquired by automatic microporous gas adsorption analyzer system on ASAP 2020 PLUS (Micromeritics, Norcross, GA, USA).

## Figures and Tables

**Table 1:** The BET parameters of CAs and AlCAs.

as	BET Surface /m^2^ g^−1^	Adsorption Average Pore Diameter /nm	Quantity Adsorbed /(cm^3^ g^−1^ STP^−1^)
AlCA–600	291.9096	2.9517	150.4746
AlCA–800	356.1491	4.9803	286.6782
AlCA–1000	292.8679	3.0761	160.2679
CA–600	306.9739	2.6727	148.4311
CA–800	287.8915	3.3322	170.8282
CA–1000	300.4066	2.4997	133.4855

**Table 2:** Density of AlCAs.

Aerogels	Thickness /mm	Diameter /mm	Weight /g	Density /g cm^−3^
AlCA–600	0.875	10	0.0157	0.229
AlCA–800	1.78	10.14	0.0401	0.279
AlCA–1000	0.519	10	0.0119	0.294

**Table 3:** Mathematical models.

Computational Domain	Conservation Equation
Mainstream	The continuity equation: $\frac{\partial \rho }{\partial t}+\frac{\partial }{\partial x_{i}}\left(\rho u_{i}\right)=S_{m}$ The momentum equation: $\frac{\partial }{\partial t}\left(\rho u_{i}\right)+\frac{\partial }{\partial x_{i}}\left(\rho u_{i}u_{j}\right)=-\frac{\partial p}{\partial x_{i}}+-\frac{\partial \tau }{\partial c_{j}}+\rho g_{i}+F_{i})$ The *SST k-ω* turbulence model was used to solve the Reynolds stress term The energy equation: $\frac{\partial }{\partial t}\left(\rho E\right)+\frac{\partial }{\partial x_{i}}\left(u_{i}\left(\rho E+p\right)\right)=\frac{\partial }{\partial x_{i}}\left(k_{f}\frac{\partial T}{\partial x_{i}}\right)+\sum_{j}h_{j}J_{j}+u_{j}{\left(\tau_{ij}\right)}_{f}+S_{h}$
Solid wall	Fourier’s law of heat conduction: $\frac{\partial }{\partial t}\rho h+\frac{\partial }{\partial x_{i}}\left(u_{i}\rho h\right)=\frac{\partial }{\partial x_{i}}\left(k_{s}\frac{\partial T}{\partial x_{i}}\right)$
Thermodynamic model	Ideal gas law: $P=\rho R_{g}T$ Sutherland formula: $\frac{\mu }{\mu_{0}}={\left(\frac{T}{T_{0}}\right)}^{1.5}\frac{T_{0}+T_{s}}{T+T_{s}}$

## Data Availability

The data that support the findings of this study are available from the corresponding author upon reasonable request.

## Nomenclature

k	Conductivity [W·m^−1^·K^−1^]
T	Temperature [K]
u	Velocity [m·s^−1^]
P	Pressure [Pa]
h	Coefficient of heat transfer [W·m^−2^·K^−1^]
R_g_	Universal or ideal gas constant [J·kg^−1^·K^−1^]
Greek	
μ	Dynamic viscosity [N·s·m^−2^]
ρ	Fluid density [kg·m^−3^]
τ	Shear stress
Subscripts	
s	Solid
*f*	Fluid
